# Dramatic Response of an Aggressive Chest Wall Recurrence to Hyperthermia, Radiation, and Chemotherapy

**DOI:** 10.7759/cureus.1479

**Published:** 2017-07-16

**Authors:** Justin Cohen, Stephanie Rice, Tejan Diwanji, Steven J Feigenberg, Zeljko Vujaskovic

**Affiliations:** 1 Department of Radiation Oncology, University of Maryland Medical Center; 2 Department of Radiation Oncology, University of Maryland School of Medicine

**Keywords:** hyperthermia, hyperthermia and chemotherapy, chest wall recurrence, hyperthermia and chest wall recurrence, non-invasive hyperthermia, localized hyperthermia and external beam x-irradiation

## Abstract

Hyperthermia has been demonstrated to be an effective adjuvant oncological treatment modality in combination with chemotherapy and/or radiation. Published data have demonstrated that the addition of hyperthermia can improve local control for breast cancer chest wall recurrences. We present a patient with a very aggressive estrogen receptor-negative, progesterone receptor-negative, HER2/neu receptor-negative chest wall recurrence status-post a right modified radical mastectomy. Despite having metastatic disease, in an attempt to achieve local control and provide palliation, she was treated with hyperthermia, radiation, and chemotherapy. A near complete resolution of her chest wall recurrence in a very short time period was seen with a significant improvement in her symptoms. While she unfortunately succumbed to her disease shortly thereafter, the local control that our treatment offered her allowed her quality of life to improve significantly near the end of her life.

## Introduction

Chest wall recurrences are a source of significant morbidity for breast cancer patients. Bleeding, ulceration, extreme pain, and psychological distress are common. Though many patients who present with a chest wall recurrence already have distant metastatic disease, achieving local control for palliation should be attempted if possible. Moderate hyperthermia (40°C - 43°C) has been demonstrated to be a chemo and radiosensitizer in *in vitro* and *in vivo *studies. Among other cancer types, there is evidence that hyperthermia can be used to improve local control in combination with radiation and/or chemotherapy for chest wall recurrences.

## Case presentation

A 38-year-old female with an unremarkable past medical and family history presented to the emergency department (ED) with a lump in the upper outer quadrant of her right breast. She admitted to noting this lump one year prior to presentation. Ultrasound examination revealed a 2.4 x 2.0 x 2.5 cm mass, along with three enlarged right axillary lymph nodes. A core biopsy revealed a poorly differentiated, estrogen receptor-negative, progesterone receptor-negative, HER2/neu-negative (triple negative), infiltrating ductal carcinoma (IDC) with a Ki-67 of 80%. Biopsy of the right axilla was negative for disease, although this was felt to be a false negative. A bone scan and computerized tomography (CT) scan of the chest, abdomen, and pelvis with contrast were negative for signs of distant metastatic disease, making her initial clinical stage T2N1M0 Stage IIB IDC.

After multidisciplinary review, she began neoadjuvant chemotherapy with Adriamycin and Cytoxan prior to surgical resection due to her triple negative disease and high proliferation index. She was able to complete three cycles but failed to follow-up for her fourth cycle. She presented back to the surgical oncologist, and on a review of her imaging, it was noted that there had been some shrinkage of the tumor initially. However, due to concern for growth after cycle 3, surgical resection was planned. She underwent breast cancer (BRCA) genetic testing, which was negative, so she proceeded with a right modified radical mastectomy with axillary dissection. The pathology revealed an 8 cm mass with two out of four nodes positive for metastatic disease with extracapsular extension (ECE). Distance from the closest margin was 1 mm. The final pathologic staging was pT3N1a.  

After sufficient healing, further adjuvant chemotherapy was planned. However, nine weeks following surgery, she presented to her surgeon with a swelling of the superior flap, first appearing five to six days prior and initially feeling like a “bump”. While this new swelling enlarged over the five to six days prior to presentation, the overlying skin had become shiny and the area had become tender. On physical exam, she was noted to have a 3 cm well-circumscribed mass, which was warm, erythematous, and extremely tender to palpation. An ultrasound revealed a 2.4 cm complex heterogeneous mass with no drainable collection. There was a concern for this being a chest wall recurrence; however, owing to the very short time period that it took to arise, it was thought to be infectious. Therefore, the patient was given a one week trial of cephalexin and instructed to follow-up to evaluate whether the swelling had responded to the antibiotics or whether further investigation was warranted.  

One week later, she presented back with no improvement in her symptoms. Ultrasound was repeated with no apparent change. An incisional biopsy revealed a recurrence of her cancer. After this resection, the surgeon felt he could not obtain negative margins and referred her to radiation oncology.   

**Figure 1:**
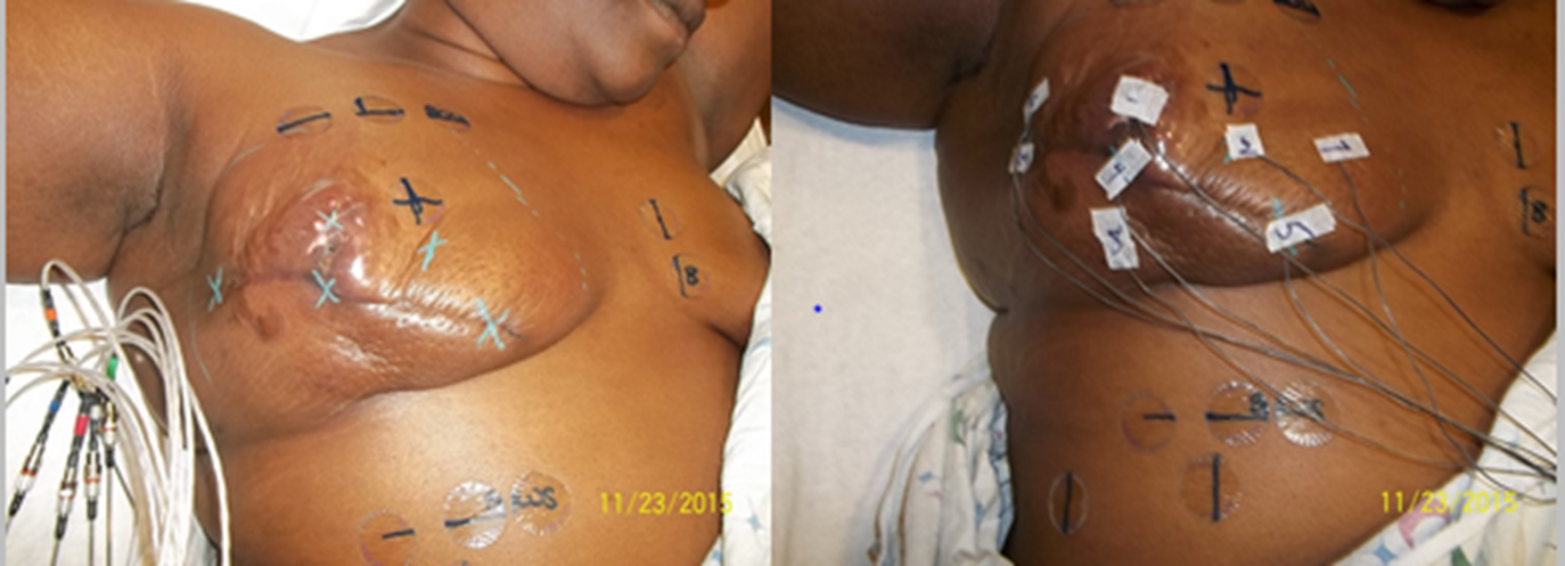
Simulation summary images of chest wall recurrence at time of treatment initiation shows skin discoloration and multiple satellite nodules This mass lesion grew aggressively between surgical resection and surgical follow-up.

**Figure 2:**
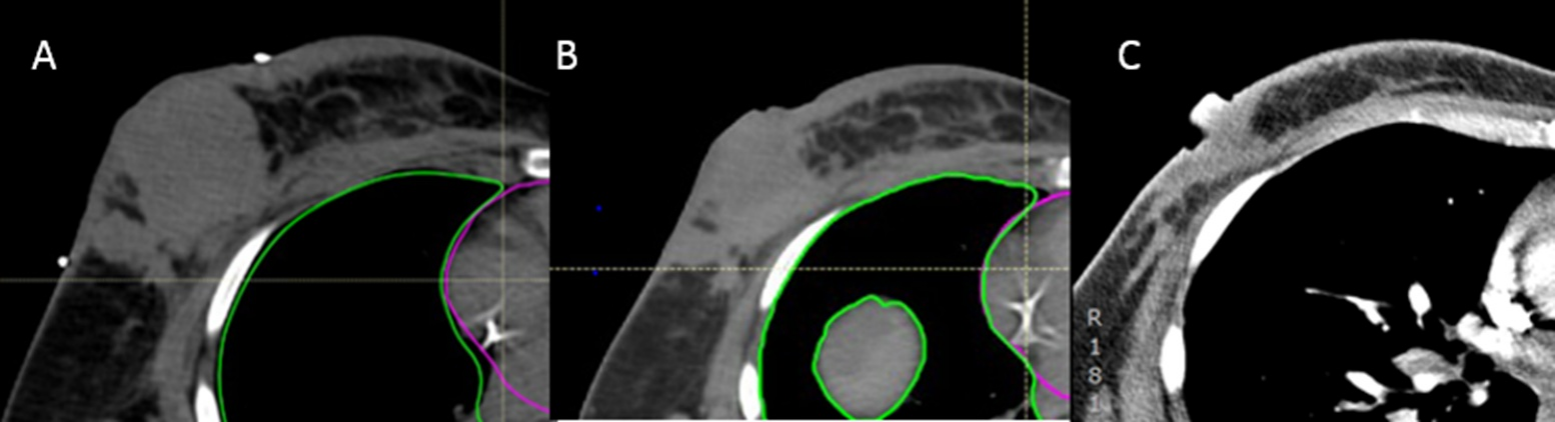
Representative images of chest wall mass A) CT simulation, B) three weeks into treatment, and C) three weeks after treatment. She developed significant pain relief by the time image B was obtained and had almost complete regression of her tumor (total < 2 cm) at the time of her diagnostic CT scan three weeks after treatment.

She presented to a radiation oncologist approximately three weeks after her chest wall recurrence (Figure 1). She complained of worsening right-sided breast pain, which she rated as 7/10 in severity, with radiation to her axilla and down to her fingertips. She was taking hydrocodone, Dilaudid, and Lyrica without any relief. On CT simulation, the mass measured 6 cm in longitudinal dimension with multifocal disease throughout the breast (Figure 2). A chest, abdominal, and pelvic CT performed five days later showed an increase in the size of the mass to 6 x 5.7 cm, multifocal and involving the pectoralis musculature, as well as noting mediastinal, hilar and subcarinal nodal disease, along with liver metastases. 

For local control and palliation of her symptoms, the initial plan was to proceed with radiation to a dose of 70 Gray (Gy) in 35 fractions (2 Gy/fraction) to the gross chest wall disease with a simultaneous integrated boost of 56 Gy in 35 fractions (1.6 Gy/fraction) to the right chest wall, axillary, supraclavicular, and internal mammary lymph nodes. However, when metastatic disease was seen, the plan was brought back to the multidisciplinary discussion where we decided to add capecitabine as opposed to offering a more abbreviated radiation schedule. As published studies have demonstrated an improvement in local control when hyperthermia is added for chest wall disease [1-2], she was treated with external thermal therapy (ETT) twice weekly to the recurrence.

Shortly after initiating treatment, she developed back pain. A magnetic resonance imaging (MRI) scan and bone scan revealed metastatic lesions to the thoracic spine at T6 and the right posterior 10th rib, which were then treated with 1 fraction of 8 Gy which she tolerated well.

Already by the third fraction of her chest wall and comprehensive nodal irradiation, she had a dramatic improvement in pain at the site of the chest wall recurrence. Her chest wall pain substantially decreased between weeks 2 and 3, at which time she started to develop radiation dermatitis. She was started on Silvadene for skin breakdown, and her pain became associated with skin irritation rather than the mass, which continuously decreased in size during the course of radiation treatments. Figure 2 demonstrates the continual decrease in the size of her chest wall mass during her treatment course. She was initially tolerating treatment fairly well, but due to the development of skin desquamation and Grade 3 dermatitis, treatment was stopped after receiving 58 Gy and completing 12 of the 14 planned hyperthermia treatments.

Though already metastatic, there had been dramatic treatment response to the chest wall recurrence. In total, her overall tumor burden decreased to < 2 cm from 9 cm with multifocal disease at the time of the CT simulation. Clinically, her tumor regressed significantly throughout treatment, and she tolerated this treatment well with the expected skin reaction, given her daily bolus to ensure adequate skin dosing.

Approximately three weeks following completion of treatment, she presented to the emergency department with a two to three-week history of worsening shortness of breath, chest, and abdominal pain. Review of her breast imaging showed significant breast tumor response with the tumor now in a single focus measuring < 2 cm with much less skin thickening and no obvious involvement of the pectoralis musculature. Unfortunately, the scan also showed diffuse bony and lung metastatic disease. She was septic with severe lactic acidosis. Soon after presentation, her status worsened and she succumbed to her disease (see Table 1 for a timeline of events).

**Table 1:** Timeline of Events IDC: invasive ductal carcinomas; CT: computed tomography; CWR: chest wall recurrence; MRI: magnetic resonance imaging; ED: emergency department; SOB: shortness of breath

Time	Reason for presentation/symptoms	Diagnosis	Treatment	Outcome
Initial presentation	Presented with lump in right breast	Poorly differentiated IDC, T2N1		
7 months after initial presentation			Completed 3 out of 5 planned cycles of neoadjuvant chemo	Initial shrinkage but growth after 3^rd^ cycle, planned for surgery
9 months after initial presentation		pT3N1a	Right modified radical mastectomy with axillary dissection	Non-concerning organizing hematoma on inferior flap, otherwise normal postop recovery
9 weeks postop	Six-day history of a growing tender mass on superior flap	Thought to be infectious	One-week course of Keflex	No shrinkage of mass and worsening local tenderness
11 weeks postop		Incisional biopsy showing recurrence		
12 weeks postop	Presented to Radiation Oncology	CT simulation performed, showed 6 cm mass	Planned to proceed with 70 Gy to gross chest wall disease and 56 Gy to the right chest wall and comprehensive nodes and concurrent hyperthermia	
13 weeks postop	Diagnostic CT to rule out metastatic disease	Mediastinal, hilar, and subcarinal nodal disease, along with liver metastases	Started on Capecitabine	
14 weeks postop			Initiated radiotherapy and hyperthermia treatments	Shrinkage of CWR to less than 2 cm, improvement pain associated with CWR
15 weeks postop	Developed back pain	MRI and bone scans reveal spine and rib metastatic lesions	1 fraction of 8 Gy to lesions	Palliation of back pain
19 weeks postop			Stopped treatment after 58 Gy and 12/14 hyperthermia treatments to the breast due to dermatitis and skin desquamation	
23 weeks postop	Presented to ED with SOB, chest, and abdominal pain	CT reveals diffuse mets		Died from respiratory failure 3 days after presentation to ED

## Discussion

There are multiple biological mechanisms behind hyperthermia-induced chemo and radiosensitization. One is that hyperthermia induces vasodilation, which results in more blood and oxygen delivery to the otherwise hypoxic regions of the tumor. This enhances the tumoricidal effect of chemotherapy and radiation. The increase in perfusion also results in increased chemo delivery to the tumor [3]. Additionally, hyperthermia impairs deoxyribonucleic acid (DNA) repair proteins, thus preventing tumor cells from repairing their DNA following sub-lethal doses of chemo and radiation. Hyperthermia has also been shown to have direct cytotoxic effects, inducing apoptosis in tumor cells - even in the absence of chemo and radiation. There is also evidence that hyperthermia augments an enhanced immune response against tumor cells. Additionally, hyperthermia makes the otherwise radioresistant S phase of the cell cycle radiosensitive. Hyperthermia can also inhibit tumor angiogenesis by preventing the expression of vascular endothelial growth factor (VEGF) [4].

The two ways that moderate hyperthermia can be delivered to superficial tumors is through capacitive heating and electromagnetic radiative heating.  In capacitive heating, the patient lies on a treatment table that has an embedded electrode. A second electrode is placed on the patient at the site of the superficial tumor. A current runs between the two electrodes, heating the tumor. In radiative heating, an antenna coupled to a water bolus is used to deliver microwaves to the tumor. A study that employed the use of hyperthermia treatment planning to compare capacitive and radiative heating found that radiative heating was superior to capacitive heating. They found that capacitive heating resulted in hot spots within fat, thus impeding the ability to get to a therapeutic temperature within the tumor. With radiative heating, the power absorption with fat was lower, thus minimizing treatment limiting hot spots [5].

At our institution, patients are treated with microwave radiative hyperthermia using the Food and Drug Administration (FDA) approved BSD-500 PC system (Pyrexar Medical, Salt Lake City, UT). Sessions are usually twice a week during radiation treatment, lasting 45-60 minutes. The temperature is raised as high as the patient can tolerate, with a maximum allowable temperature of 44°C (preferably in the range of 40-43°C).

The local control benefit for chest wall recurrences treated with hyperthermia is well described. In 1996, Vernon, et al. published a meta-analysis of 306 patients from five Phase III trials comparing radiation, plus hyperthermia, to radiation alone [1]. Hyperthermia increased complete response (CR) rates from 41% to 59%. A more pronounced difference was noted when a lower dose of radiation was administered due to previous irradiation (57% vs. 31%). Thermal blistering was observed in 11% of patients. In 2005, Jones, et al. published data from a Phase III trial of 109 patients with superficial tumors, randomized to receive hyperthermia, plus radiation, or radiation alone [2]. In patients who were not previously irradiated, in the hyperthermia arm, 65% had a CR whereas, in the radiation alone arm, 51% had a complete response. The difference between the two treatment arms was even more pronounced in previously irradiated patients. In the hyperthermia arm, 68% of patients had a CR whereas, in the radiation alone arm, 24% had a complete response. The majority of patients enrolled in this study had chest wall recurrences. Hyperthermia was generally well tolerated. There were 26 burns among 56 patients (46%) who received 600 hyperthermia treatments (4.3%). The majority of these thermal burns were first degree.  

Since some chest wall recurrences happen in the setting of metastatic disease, studies have not demonstrated an overall survival (OS) benefit to hyperthermia. However, the morbidity associated with local recurrence warrants consideration of its use for local control benefit. Our patient had a very aggressive recurrence causing significant pain and distress. Metastatic disease was identified prior to initiating therapy. Nonetheless, she was treated with radiation, chemotherapy, and hyperthermia, which resulted in a near complete resolution of her chest wall recurrence in a very short time period and a significant improvement in her symptoms. While she did succumb to her disease, the local control that our aggressive multimodality treatment offered her allowed her quality of life to improve significantly near the end of her life. This clinical endpoint is incredibly significant for patients with terminal disease, and improvements in quality of life should be emphasized in these patients.

## Conclusions

A 38-year-old female presented to our department with an aggressive chest wall recurrence, as well as with distant metastatic disease, two months following a right modified radical mastectomy for a pathological T3N1a breast cancer. This recurrence was causing extreme pain and distress. As clinical trials have demonstrated an improvement in local control when hyperthermia is used as an adjunct to radiation therapy, our patient was treated with radiation, chemotherapy, and hyperthermia. Local control was achieved, providing her with palliation of her symptoms. This case underscores the importance of considering hyperthermia as part of a multimodality treatment regimen to treat chest wall recurrences.
